# Editorial: Emerging Roles for Type 2-Associated Cells and Cytokines in Cancer Immunity

**DOI:** 10.3389/fimmu.2021.811125

**Published:** 2021-11-26

**Authors:** Giovanna Schiavoni, Ariel Munitz, Jessica Strid

**Affiliations:** ^1^ Department of Oncology and Molecular Medicine, Istituto Superiore di Sanità, Rome, Italy; ^2^ Department of Clinical Microbiology and Immunology, Tel Aviv University, Tel Aviv, Israel; ^3^ Department of Immunology and Inflammation, Imperial College London, London, United Kingdom

**Keywords:** cancer immunity, type 2 associated cells, Th2 cytokines, tumor microenvironment, granulocytes

Type 2 immune responses have mainly been studied in the context of parasite infections and allergic diseases. However, emerging evidence suggests important roles for Type 2 immunity in multiple additional physiological and pathological settings (1). In fact, Type 2-associated cells, such as eosinophils, mast cells, basophils, Type 2 innate lymphoid cells (ILC2), as well as the cytokines IL-4, IL-5, IL-13, IL-33 and thymic stromal lymphopoietin (TSLP) are now considered a significant signaling and effector cell axis in metabolism, tissue remodeling, neuroinflammation and cancer. This Research Topic collects relevant studies on the emerging role of some Type 2-associated cells and cytokines in the modulation of cancer progression and anti-tumor immune responses.

Among the Type 2 cytokines, IL-33 and TSLP are epithelial-derived alarmins capable of activating a plethora of innate and adaptive immune cell populations, thereby affecting tumor immune control. Some Type 2-associated cells, such as mast cells, eosinophils and basophils, display pleiotropic activities within the tumor microenvironment (TME). On the one hand, they can provide growth and angiogenic factors to promote tumor growth. On the other hand, they can exert tumor cytotoxicity through degranulation and release of soluble mediators. In a mini review article, Eissmann et al. debate the role of the IL-33/ST2 axis and mast cells in the regulation of gastrointestinal cancer. Mast cells can regulate IL-33 bioactivity through the release of tryptase and chymase that cleave the immature full-length form of IL-33 into the mature form of the cytokine. Several immune cells, including mast cells, express ST2 at various levels on their cells surface and respond to IL-33 in the TME of gastrointestinal cancers. During pre-cancerous inflammation, a functional cooperation between IL-33 and mast cells promotes the restoration of the epithelial barrier and the regeneration of gastrointestinal epithelium. In colorectal cancers however, albeit the expression of IL-33 and the prevalence of mast cells are not necessarily associated, both appear to have a tumor-promoting role in most pre-clinical and human studies. In gastric cancers, the authors discuss an interesting study involving an IL-33/mast cell/macrophage axis that promotes tumor growth. Here, activation of mast cells by IL-33 resulted in the secretion of macrophage attracting factors and subsequent accumulation of pro-angiogenic and pro-tumorigenic macrophages in the gastric tumors. Finally, the authors discuss the possibility of harnessing the IL-33/mast cell axis taking into account the dichotomous role they play in gastrointestinal tumors and other cancers.

IL-33 is a pleiotropic cytokine that can activate both innate and adaptive immune cells (and stimulate both Th1 and Th2 responses), which result in dichotomous roles in tumor immunity, clearly depending on the tumor type and the nature of the TME. In another mini review article, Andreone et al. focus on the anti-tumoral mechanisms exerted by IL-33, *via* stimulation of several different immune effector cells. Recent literature indicates that IL-33 can sustain the effector activity of CD8^+^ T cells and NK cells and promote the functions of CD4^+^ T lymphocytes in the TME. Moreover, IL-33 can activate the cytotoxic functions of eosinophils and basophils promoting tumor killing. In addition, IL-33/ST2 stimulation enhances the anti-tumor functions of ILC2 cells through multiple mechanisms, including recruitment of eosinophils and cross-presenting DCs, ultimately resulting in tumor cytotoxicity. Finally, recent evidence demonstrating that IL-33 can modulate the expression of immune checkpoints (i.e., PD-1/PD-L1 and CTLA-4) in certain immune cells opens perspectives for targeting the IL-33/ST2 axis to increase immunotherapy efficacy. This is of specific interest as although immune checkpoint inhibitors are one of the most promising avenues in cancer immunotherapy only a subset of patients demonstrate a clinical response. Therefore, it is critically important to identify predictive biomarkers for a beneficial response to ICI therapy and to understand the role of all immune cells and soluble mediators in cancer immunotherapy.

Another epithelial-derived alarmin, TSLP, is a lymphopoietin commonly expressed in the skin, gut and lung tissues. Expression of TSLP and/or its receptor is found in several human cancers and may be associated with the induction of a Type 2-prone TME. In a review article, Protti and De Monte debate Th2-dependent and Th2-independent roles of TSLP in several cancer types and the possible therapeutic targeting of this cytokine. Several studies on human and mouse tumors found either pro- or anti-tumor activity of TSLP, mostly based on the association between TSLP expression and the development of predominant Th2 inflammation in the tumor, or direct TSLP signaling on tumor cells. Protti and De Monte propose a model by which TSLP released by tumor cells and cancer-associated fibroblasts can activate Type 2 immunity to foster cancer progression *via* myeloid TSLP-expressing DCs and M2 macrophages in the stroma, which ultimately allow TSLP-DCs to activate Th2 pro-tumoral responses in the tissue draining lymph node. In view of the pro-tumor *vs*. antitumor function of TSLP in different tumor types, its possible manipulation for therapeutic purposes will need further investigation.

Finally, in a detailed review, Marone et al. discuss the functional characteristics of mouse and human basophils, and how these may dictate tumor fate. Basophils are a Type 2-associated cell subset whose role in cancer is only beginning to be studied. They are a rare immune cell subset representing <1% of human peripheral blood leukocytes but they can accumulate in inflamed tissues and possess powerful effector mechanisms. Basophils can modulate cancer progression through the production of a plethora of angiogenic factors (e.g., VEGF-A, VEGF-B, HGF, ANGPT1 and CXCL8). Furthermore, basophils can release DNA extracellular traps (ETs), which have an antibacterial function but may also promote metastasis and cancer-associated thrombosis, as described for neutrophil ETs. Basophils can also produce a variety of cytokines (e.g., IL-3, IL-4, IL-6 and IL-13) and display plasticity of phenotype and function under the influence of the TME. In addition, under appropriate stimulation, basophils can acquire tumoricidal properties *in vitro*. The generation of genetically engineered mouse models has allowed studying the functional role of basophils in pancreatic ductal adenocarcinoma (PDAC) *in vivo*. Here, the authors propose a model in which basophils recruited to the tumor-draining lymph nodes (TDLN) skew towards Th2 and M2 polarization through the production of IL-4 and by this mechanism play a relevant pro-tumorigenic role in PDAC progression. Overall, this review underlines that despite the established presence of basophils in human and experimental cancers, further investigation is required to elucidate the role of basophils in tumor immunity.

In sum, it is becoming increasingly clear that Type 2-associated cells and soluble mediators are an important part of the TME and can be central orchestrators of cancer development. The published articles in this Research Topic aim to provide a better understanding of the mechanisms operated by Type 2 immune effectors and their interplay with other components of the TME in specific tumor types. We hope this collection will inspire future work exploring and harnessing these overlooked immune components for future therapeutic strategies against cancer.

## Author Contributions

GS wrote the editorial and invited authors to contribute the Research Topic. AM co-wrote the editorial and invited authors to contribute to the Research Topic. JS co-wrote the editorial and invited authors to contribute to the Research Topic. All authors contributed to the article and approved the submitted version.

## Funding

GS is supported by AIRC (IG 21366). AM is supported by the US-Israel Bi-national Science Foundation (grant no. 2015163), by the Israel Science Foundation (grants no. 886/15 and 542/20), the Israel Cancer Research Fund, the Richard Eimert Research Fund on Solid Tumors (TAU), the Israel Cancer Association Avraham Rotstein Donation, the Cancer Biology Research Center (TAU) and the Emerson Collective. JS is supported by the Wellcome Trust (215488/Z/19/Z) and the British Skin Foundation (043/S/18).

## Conflict of Interest

The authors declare that the research was conducted in the absence of any commercial or financial relationships that could be construed as a potential conflict of interest.

## Publisher’s Note

All claims expressed in this article are solely those of the authors and do not necessarily represent those of their affiliated organizations, or those of the publisher, the editors and the reviewers. Any product that may be evaluated in this article, or claim that may be made by its manufacturer, is not guaranteed or endorsed by the publisher.
